# TOC-PAT: a novel approach to discharge stewardship

**DOI:** 10.1017/ash.2026.10852

**Published:** 2026-09-23

**Authors:** Rebecca Lee, Karan Raja, Soo Kang, Mitesh Patel, Mona Philips

**Affiliations:** 1 Clara Maass Medical Center, USA; 2 Rutgers, The State University of New Jersey, New Brunswick, NJ, USA

## Abstract

**Background:**

Inpatient antimicrobial stewardship (AMS) programs combat inappropriate antibiotic use, increased resistance, and adverse outcomes. However, stewardship monitoring is limited at transitions of care (TOC), especially for patients being discharged on parenteral antimicrobials. Additionally, discharge planning processes for these patients are often complex, prolonging length of stay (LOS). This highlights an opportunity to optimize outpatient parenteral antimicrobial therapy (OPAT) regimens following inpatient admissions.

**Objective:**

To compare LOS in patients discharged with OPAT before versus after a stewardship initiative targeting TOC parenteral antimicrobial therapy (TOC-PAT).

**Design:**

Prepost cohort study.

**Setting:**

Urban, non-teaching, community medical center.

**Patients:**

Adult patients discharged or planned to be discharged on OPAT.

**Methods:**

The AMS team developed a novel program monitoring patients being discharged on OPAT. Pharmacists were deployed to identify AMS intervention opportunities. This study evaluated outcomes before and after program implementation. The primary outcome compared inpatient LOS. The secondary outcome assessed antimicrobial appropriateness using the National Antimicrobial Prescribing Survey.

**Results:**

The pre- and postimplementation cohorts included 167 and 100 patients, respectively. There was no statistically significant difference in median LOS (9 [6, 13] vs 10 [6,15] days; *P* = .27). The proportion of appropriate regimens increased from 51.5% to 58%. A total of 68 pharmacist-led AMS interventions were made, 66% of which were accepted by treating teams, primarily modifications in antimicrobial choice and IV to PO conversions.

**Conclusion:**

This study serves as a foundation for future OPAT and TOC-PAT stewardship programs. The results of this study highlight pharmacist impact in assessing OPAT regimens to increase antimicrobial appropriateness.

## Introduction

Inappropriate antimicrobial prescribing is associated with increased treatment failure, adverse drug events, antimicrobial resistance, inpatient length of stay (LOS), and associated costs.^1,2^ Antimicrobial stewardship (AMS) programs are hospital or community-based programs that measure and improve antimicrobial prescribing by clinicians and use by patients. Since the early 2010s, there has been continued emphasis toward improving AMS efforts in the inpatient setting, but there are limited AMS resources at hospital discharge and transitions of care (TOC). Many patients admitted to the hospital for management of infectious disease are continued on parenteral antimicrobial therapy at discharge, despite increasing evidence suggesting that oral antimicrobials are comparable to parenteral regimens for most indications.^3–7^ Studies have shown that up to 70% of antimicrobial prescriptions are inappropriate at hospital discharge, therefore identifying a potential opportunity for targeted TOC stewardship efforts.^8,9^ With this being said, pharmacists are well-positioned to support AMS at TOC through prospective audit and feedback interventions, critical appraisal of pharmacotherapy, and navigating social, insurance, and financial barriers in collaboration with the treatment teams. Pharmacist-led AMS initiatives demonstrate high intervention acceptance rates associated with more optimal regimens, fewer antimicrobial-related adverse events, reduced hospital LOS, and increased healthcare cost savings.^10–14^ One study found that TOC pharmacists optimized the dose, frequency, duration, and/or antimicrobial choice in 48.9% of OPAT prescriptions, underscoring the impactful role that pharmacists can have in this space.^13^

A previous study conducted at our institution evaluated appropriateness of oral antimicrobial regimens prescribed at discharge.^14^ The gap analysis found that 53% of these oral antimicrobial regimens were inappropriate due to drug choice, dosing, and/or duration. In light of these findings, the AMS program implemented a multidisciplinary, multifaceted quality improvement initiative, comprised of development of a comprehensive, institution-specific antimicrobial prescribing guideline, face-to-face clinician education, and real-time pharmacist prospective audit and feedback at discharge. Antimicrobial appropriateness increased by 15% (47% vs 62%, *P* = .03). Based on these findings, the discharge stewardship program was expanded to include review of parenteral antimicrobials, in collaboration with hospital administration, case management, pharmacy, and medicine. The program goal was to increase OPAT appropriateness and streamline the disposition planning process, which is often prolonged in the process of reconciling OPAT treatment plans. The objective of this study was to assess the impact of this expanded, novel TOC-PAT program by comparing the inpatient LOS before versus after implementation.

## Methods

This was a single-center, prepost design study approved by the Institutional Review Board and Institutional Review Committee with receipt of a waiver of informed consent. This study took place at a 472-bed, urban, acute care, non-teaching, community medical center part of a larger health system in the United States. Interdisciplinary discharge rounds (IDR) take place daily to evaluate care plans and identify barriers to discharge for all inpatients, including OPAT plans and discharge disposition. Historically, the disciplines involved in these crucial conversations included hospitalists, case management, and nursing leadership. Therefore, this study was designed to optimize discharge planning with the integration of clinical pharmacy specialists in discharge planning to augment AMS guidance during TOC.

This study implemented a novel TOC-PAT program that consisted of the expansion of institution-specific antimicrobial prescribing guidelines, clinician education on OPAT-focused stewardship principles, and pharmacist integration in IDR to identify and intervene on direct prospective audit and feedback for planned OPAT regimens.

An institution-specific prescribing guideline was locally developed and disseminated to standardize antimicrobial prescribing across clinical disciplines in the hospital. This guideline included first-line empiric antimicrobial recommendations for the most frequently encountered infectious diseases, as well as alternative choices, therapy duration, and step-down or de-escalation strategies. To target the OPAT patient population at discharge, these guidelines were updated and expanded upon with common disease states for which outpatient infusions may be indicated, including bloodstream infections, diabetic foot infections, osteomyelitis, and endocarditis. Recommendations were supported by primary literature, national guidelines, and local susceptibility patterns.

In preparation for the initiation of direct pharmacist prospective and feedback on OPAT regimens prescribed at discharge, education was disseminated to clinicians to highlight the retrospective gap analysis findings and best practice stewardship principles for the target patient population. Printed educational material alongside verbal presentation was provided to clinicians of various specialties, including infectious diseases, intensive care, general medicine, surgical podiatry, in addition to presentation at institutional grand rounds. ID providers were the primary audience for education as they prescribe or oversee the overwhelming majority of parenteral antimicrobials prescribed at discharge at our institution.

The prospective audit and feedback component was instrumental in establishing a novel standard of practice at our institution with the introduction of clinical pharmacist presence at IDR on a daily basis. Upon identifying patients in IDR on a parenteral antimicrobial regimen with plans to continue with OPAT therapy, the pharmacist screened the patient for appropriateness and opportunities for therapy optimization, including but not limited to: de-escalation and/or transition to oral antimicrobials, and addressing compatibility of the chosen antimicrobial with the preferred discharge disposition site. The pharmacist also provided recommendations for utilization of the safest vascular access device based on the antimicrobial regimen and anticipated treatment duration in alignment with our local midline or vascular access policies and procedures. All of these interventions aimed to enhance patient safety, facilitate earlier hospital discharge, and reduce healthcare costs. If a regimen was deemed inappropriate, the pharmacist intervened by providing recommendations to the prescriber and/or medical team. If the recommendation was accepted, the pharmacist documented the intervention and communicated any relevant changes to nursing and case management, as necessary. The pharmacist also directly facilitated communications with infectious disease specialists in the setting of any barriers to OPAT identified with case management.

The preimplementation cohort included adult patients who were discharged on at least one parenteral antimicrobial from January through March of 2024. Guideline updates, plan development, and hospitalwide education occurred between November and December 2024. The postimplementation cohort included adult patients who were discharged on at least one parenteral antimicrobial from January through March of 2025. Patients were excluded if they were pregnant or postpartum.

The primary outcome was median hospital LOS. Additional outcomes included antimicrobial appropriateness, intervention acceptance rate, total antimicrobial days of therapy, vascular access at discharge, all-cause 30-day readmission rate, and discharge disposition. Antimicrobial appropriateness was evaluated using the National Antimicrobial Prescribing Survey (NAPS) a validated screening tool used to categorize antimicrobial regimens as appropriate (including optimal or adequate) or inappropriate (including suboptimal or inadequate).^15^ Regimens may also be categorized as not assessable in the setting of inadequate documentation. The survey considers antimicrobial choice, dosing, frequency, duration, and allergy mismatch in assessing compliance with guideline recommendations. The utilization of the NAPS tool has been shown to be feasible, generalizable, and translatable to various settings, with high accuracy and inter-rater reliability rates.^15,16^ It provides a simplified, standardized approach to qualifying antimicrobial appropriateness and thus has been utilized for accreditation and institutional quality improvement purposes. Two clinical pharmacists independently evaluated NAPS appropriateness of each regimen and in the case of any discrepancies, a third pharmacist made the final determination.

Additional outcomes assessed included antimicrobial duration, vascular access, discharge disposition, and 30-day readmission. Data were evaluated using t-test, χ^2^ test, and descriptive statistics.

A posthoc analysis was evaluated for the subgroup of patients for which direct pharmacist interventions were made during the study period. The acceptance rate and intervention type distribution were evaluated, as well as all other outcomes included for the pre versus post gap analysis.

## Results

In the pre and postimplementation cohorts, 167 and 100 patients were included in the analysis, respectively. There were no significant differences in the baseline characteristics between groups (Table 1). The most common indications for which OPAT was planned included genitourinary infections, bloodstream infections, bone/joint infections, and acute bacterial skin and skin structure infections.

**Table 1.** Baseline characteristics Table 1 long description.

Characteristic	Preintervention (n = 167)	Postintervention (n = 100)
Mean age—years (±SD)	66 (±16)	67 (±15)
Sex—no. (%)		
Female	84 (50.3)	43 (43)
Insurance coverage—no. (%)		
Commercial	24 (14.9)	17 (17)
Medicare	106 (63.5)	63 (63)
Medicaid	30 (17.9)	15 (15)
Uninsured	7 (4.2)	4 (4)
Other		1 (1)
Indication^a^—no. (%)		
Genitourinary	53 (31.5)	25 (25)
Bloodstream	38 (22.6)	28 (28)
Bone/Joint	36 (21.4)	20 (20)
Skin and soft tissue	34 (20.2)	22 (22)
Respiratory	15 (8.9)	10 (10)
Endocarditis	7 (4.2)	7 (7)
Intra-abdominal	6 (3.6)	6 (6)

SD, standard deviation.

a Patients may have had more than one concomitant infection or antimicrobial indication.

There was no statistically significant difference observed in median inpatient LOS pre and postintervention, at 9 [6,13] versus 10 [6,15] days (*P* = .27) (Table 2). The median total antimicrobial days of therapy, including inpatient plus outpatient, was 9 days in both groups.

**Table 2.** Results Table 2 long description.

	Pregroup (n = 167)	Postgroup (n = 100)	Posthoc group (n = 63)^a,b,c^
Primary outcome			
Length of stay, days—median [IQR]	9 [6,13]	10 [6,15]	12 [8,21]
	*P* = .27		
Secondary outcome—n (%)			
Optimal (NAPS 1)	27 (16.2)	30 (30)	12 (26.7)
Adequate (NAPS 2)	59 (35.3)	28 (28)	11 (24.4)
Suboptimal (NAPS 3)	49 (29.3)	25 (25)	18 (40)
Inadequate (NAPS 4)	16 (9.6)	5 (5)	3 (6.7)
Not assessable	16 (9.6)	12 (12)	1 (2.2)
Exploratory outcomes			
Total antimicrobial days of therapy—median [IQR]	9 [14,28.5]	9 [14,32]	14 [14,21]
Vascular access—n (%)			
Peripheral	45 (27)	24 (24)	2 (3.2)
Midline	68 (40.7)	44 (44)	7 (11.1)
Central	52 (31.1)	28 (28)	5 (7.9)
None	0 (0)	0 (0)	40 (63.5)
Not specified	2 (1.2)	4 (4)	4 (6.35)
Discharge disposition—n (%)^d^			
Home-IV	59 (35.3)	40 (40)	8 (12.7)
Facility	108 (64.7)	60 (60)	24 (38.1)
Home-no IV	0 (0)	0 (0)	31 (49.2)
30-day all-cause readmission—n (%)			
Admission	30 (17.9)	16 (16)	16 (25.3)
ED visit	17 (10.1)	12 (12)	5 (7.9)

a Excludes duplicate encounters.

b Pharmacist intervention group, which included patients that had pharmacist review (includes those discharged on oral, parenteral, or no antimicrobials).

c Patients discharged on parenteral in this group were also included in the “PostGroup”.

d “Home-IV” includes home infusion and infusion with outpatient dialysis; “Facility” includes long-term care, skilled nursing facility, long-term acute care, hospice, or other facility; “Home-no IV” includes discharge home with oral antimicrobials or no antimicrobials.

**Figure 1.**
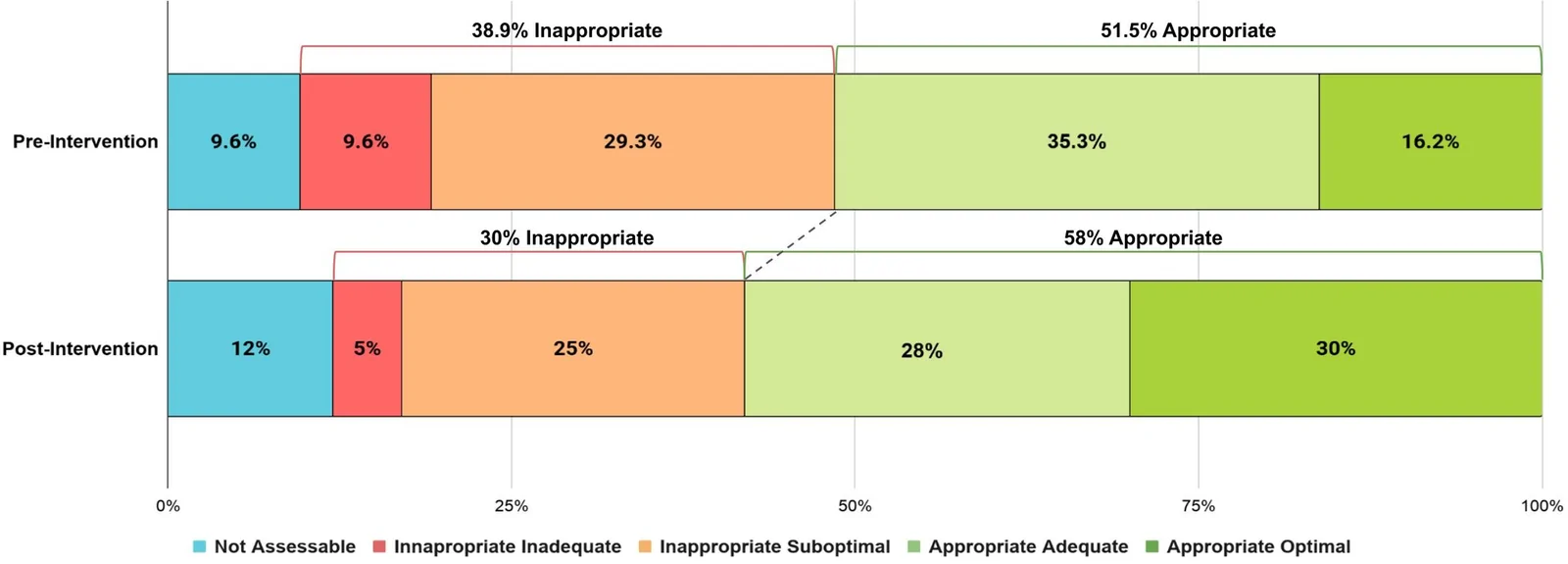
Proportion of NAPS appropriateness.

There was an increase in the proportion of appropriate OPAT regimens pre and postimplementation at 51.5% versus 58%, respectively (Figure 1). In the preimplementation cohort, the most common inappropriately prescribed antimicrobials were piperacillin-tazobactam (24.1%), cefepime (17.2%), and ceftriaxone (17.2%), and the most common inappropriately treated indications were acute bacterial skin and skin structure infections (56.7%), osteomyelitis (47.2%) and genitourinary infections (32.4%). In the postimplementation cohort, the most common inappropriately prescribed antimicrobials were piperacillin-tazobactam (21.1%), ceftriaxone (13.2%), meropenem (10.5%), and ertapenem (10.5%), and the most common inappropriately treated indications were acute bacterial skin and skin structure infections (27.8%), bloodstream infections (22.2%), and osteomyelitis (16.7%).

**Figure 2.**
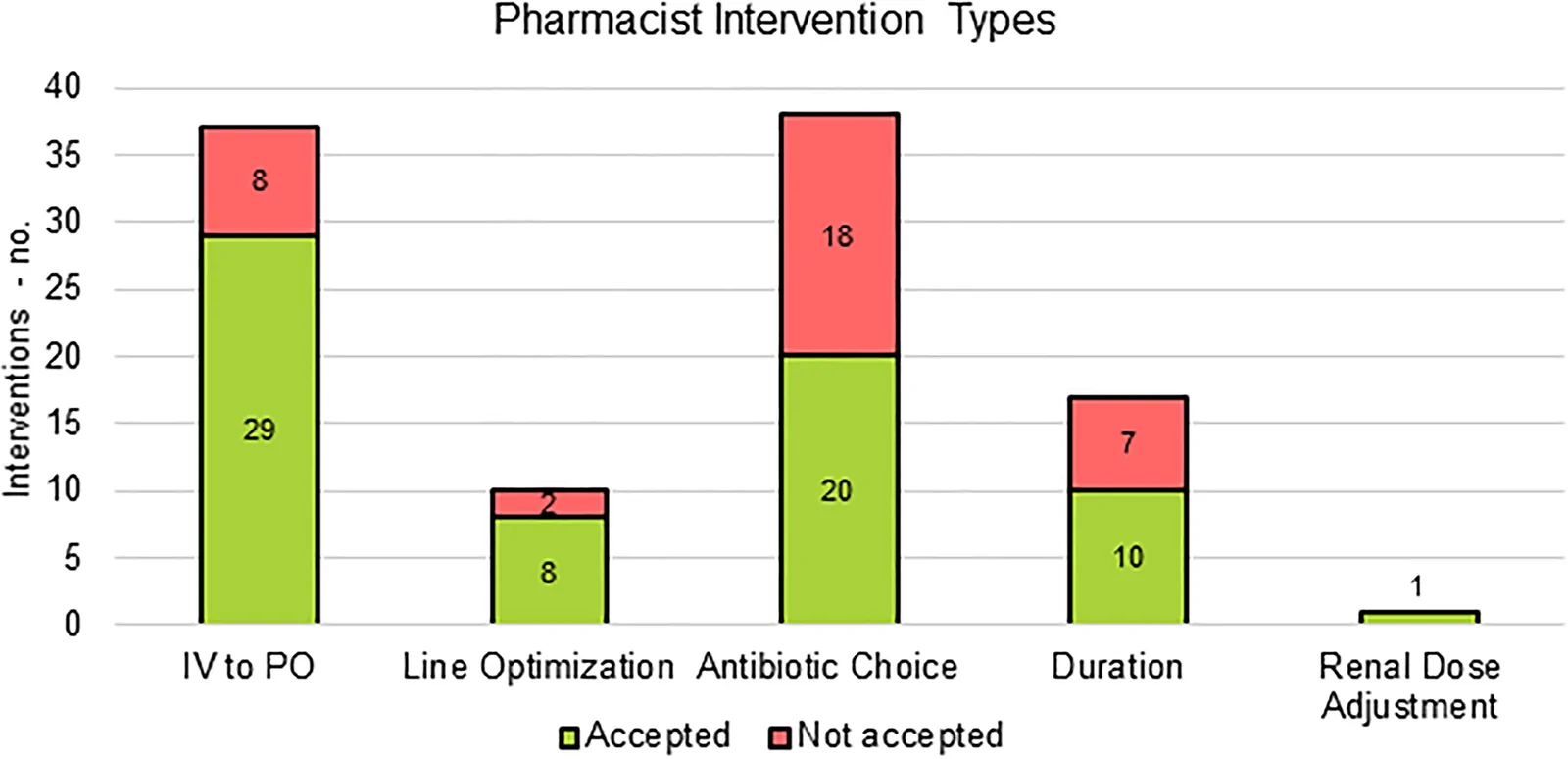
Distribution of pharmacist-driven intervention type.

Pharmacists were directly involved in the care of 63 patients during the study period, including those discharged on oral, parenteral, or no antimicrobials. A total of 68 pharmacist interventions were made at a 66% acceptance rate. The leading types of interventions made and accepted were modifications in antimicrobial choice, followed by IV to PO conversions (Figure 2 *)*. In the pharmacist intervention subgroup, 63.5% of patients were able to be discharged without parenteral access, and 49.2% of patients were sent home without IV antimicrobials, highlighting the opportunities identified to transition patients to oral antimicrobials or discontinue antimicrobial treatment altogether.

## Discussion

The implementation of a novel, multidisciplinary and multifaceted TOC-PAT initiative increased the overall proportion of appropriate OPAT regimens being prescribed at hospital discharge. Previous studies with TOC stewardship initiatives focused on oral antimicrobial regimens demonstrated improved appropriateness by up to 18%.^14,18^ In our study, the proportion of optimal regimens nearly doubled from 16.2 to 30% while the proportion of inadequate regimens was reduced from 9.6 to 5%.^8^ These improvements are reflective of the observed acceptance rate of pharmacist interventions at 66%, which is clinically meaningful but reduced compared to acceptance rates of 70% to 80% seen in previous pharmacy-led TOC stewardship program studies.^14,19^ In our study, interventions may not have been accepted for several reasons. Interventions were primarily formulated based on available clinical information, multidisciplinary discussion during IDR, and chart-review. Therefore, certain relevant, but unrecognized, patient-specific factors (eg,: socioeconomic barriers and insurance-related limitations), which may only be identified through bedside assessment or patient interview, may not have been fully captured. These additional considerations could have influenced both the recommendation and its subsequent acceptance.

This initiative highlighted opportunities for de-escalation and oral conversion to minimize the risk of vascular access complications and avoid excessive antimicrobial use. A substantial proportion of patients originally planned to be discharged on OPAT were discharged without any parenteral vascular access and without any IV antimicrobials prescribed, at 63.5 and 49.2%, respectively. Therefore, although the primary end point of inpatient LOS did not change significantly, this study emphasizes the impact that TOC-focused stewardship interventions can have on the appropriateness of parenteral antimicrobial therapy at hospital discharge. Additionally, integrating a pharmacist in the patient care planning process during TOC can improve interdisciplinary collaboration, antimicrobial medication reconciliation, and optimize outpatient antimicrobial therapy.

There is limited research on the impact of TOC-focused interventions in the OPAT stewardship space, particularly in non-teaching community hospitals without dedicated TOC or OPAT pharmacists. This study contributes to the existing literature by evaluating the impact of pharmacist-led stewardship in a novel TOC parenteral antimicrobial therapy program, targeting optimization of OPAT regimens prior to discharge, and facilitating implementation of best practice stewardship principles across disciplines. This study also evaluated several additional implicated metrics pertinent to TOC, such as vascular accesses and discharge disposition associated with pharmacist-led interventions.

Our study had several limitations. Firstly, logistical challenges precluded maximal pharmacist integration at IDR. Therefore, there was limited ability to comprehensively identify and review all patient cases with opportunity for intervention. This may have affected the ability of our study to establish a significant difference in our primary outcome. Additionally, the NAPS appropriateness tool evaluates antimicrobial regimens based on consistency with optimal guidelines. However, in many cases, socioeconomic factors played a role in deciding the antimicrobial choice during discharge disposition planning, which may have impacted appropriateness categorization. For example, a commonly utilized home infusion service partner was only able to accept once-daily antimicrobial agents for OPAT prescriptions, therefore requiring modifications to certain antimicrobial regimens during discharge planning, some of which may not have aligned with AMS best practice. Another limitation is rooted in the assumption of complete and accurate diagnoses of infectious etiologies for which the antimicrobials assessed were intended to treat. This overlooks the trend that antimicrobials are often overused and unwarranted in cases where they may not be indicated. Lastly, the lack of complete and consistent documentation in the electronic medical records presented a barrier to fully assessing and optimizing all planned OPAT regimens, consequently precluding pharmacist intervention.

Future directions include continued focus on cultural shifts toward appropriate use of oral antimicrobials for indications historically treated with parenteral antimicrobials, such as Gram-negative bacteremia and osteomyelitis.^3–7,11–12^ Additionally, reinforcement of provider education for complete documentation of key clinical characteristics, such as bone debridement margins in the context of osteomyelitis, which would impact appropriate antimicrobial plan. Such interventions may influence LOS outcomes, presenting substantial cost avoidance opportunities. The results of this study highlight pharmacist impact as a part of an AMS team on LOS, antimicrobial appropriateness, discharge disposition, and vascular access optimization when integrated into TOC-focused stewardship initiatives.

## Data Availability

Access to data may be provided upon reasonable request.

## Table 1. Long description

A table comparing baseline characteristics of patients in preintervention and postintervention cohorts. The table has 12 rows and 4 columns. The columns are labeled ʼCharacteristicʼ, ʼPreintervention (n = 167)ʼ, and ʼPostintervention (n = 100)ʼ. The rows include characteristics such as mean age, sex, insurance coverage, and indication. For mean age, the preintervention group has a mean age of 66 years with a standard deviation of 16, while the postintervention group has a mean age of 67 years with a standard deviation of 15. For sex, 84 females (50.3 percent) are in the preintervention group, and 43 females (43 percent) are in the postintervention group. Insurance coverage includes categories like Commercial, Medicare, Medicaid, Uninsured, and Other, with varying percentages in each group. Indications include categories like Genitourinary, Bloodstream, Bone/Joint, Skin and soft tissue, Respiratory, Endocarditis, and Intra-abdominal, with percentages provided for each indication in both groups.

Navigate back to Table 1..

## Table 2. Long description

A table comparing outcomes of different groups in a medical study. The table has 13 rows and 4 columns. The columns are labeled Pregroup (n = 167), Postgroup (n = 100), and Posthoc group (n = 63). The rows are labeled with various outcomes and their respective values. Row 1: Length of stay, days—median [IQR], Pregroup 9 [6,13], Postgroup 10 [6,15], Posthoc group 12 [8,21]. Row 2: Optimal (NAPS 1), Pregroup 27 (16.2), Postgroup 30 (30), Posthoc group 12 (26.7). Row 3: Adequate (NAPS 2), Pregroup 59 (35.3), Postgroup 28 (28), Posthoc group 11 (24.4). Row 4: Suboptimal (NAPS 3), Pregroup 49 (29.3), Postgroup 25 (25), Posthoc group 18 (40). Row 5: Inadequate (NAPS 4), Pregroup 16 (9.6), Postgroup 5 (5), Posthoc group 3 (6.7). Row 6: Not assessable, Pregroup 16 (9.6), Postgroup 12 (12), Posthoc group 1 (2.2). Row 7: Total antimicrobial days of therapy—median [IQR], Pregroup 9 [14,28.5], Postgroup 9 [14,32], Posthoc group 14 [14,21]. Row 8: Vascular access—Peripheral, Pregroup 45 (27), Postgroup 24 (24), Posthoc group 2 (3.2). Row 9: Vascular access—Midline, Pregroup 68 (40.7), Postgroup 44 (44), Posthoc group 7 (11.1). Row 10: Vascular access—Central, Pregroup 52 (31.1), Postgroup 28 (28), Posthoc group 5 (7.9). Row 11: Vascular access—None, Pregroup 0 (0), Postgroup 0 (0), Posthoc group 40 (63.5). Row 12: Vascular access—Not specified, Pregroup 2 (1.2), Postgroup 4 (4), Posthoc group 4 (6.35). Row 13: 30-day all-cause readmission—Admission, Pregroup 30 (17.9), Postgroup 16 (16), Posthoc group 16 (25.3). Row 14: 30-day all-cause readmission—ED visit, Pregroup 17 (10.1), Postgroup 12 (12), Posthoc group 5 (7.9).

Navigate back to Table 2..
